# Somatostatin Receptor Avidity in Gastrointestinal Stromal Tumors: Theranostic Implications of Gallium-68 Scan and Eligibility for Peptide Receptor Radionuclide Therapy

**DOI:** 10.7759/cureus.1710

**Published:** 2017-09-24

**Authors:** Arturo Loaiza-Bonilla, Paula A Bonilla-Reyes

**Affiliations:** 1 Medicine, Hematology and Oncology, Cancer Treatment Centers of America; 2 Facultad de Medicina, Pontificia Universidad Javeriana

**Keywords:** gallium-68, gastrointestinal stromal tumor, gist, peptide receptor radionuclide therapy, prrt, somatostatin receptor, personalized oncology, sarcoma, nuclear medicine, biomarkers

## Abstract

This manuscript reports on a patient with a metastatic gastrointestinal stromal tumor (GIST) refractory to standard first-line treatment, who underwent a gallium-68 scan based on pre-clinical data of somatostatin receptor (SSTR) expression in such tumors. The gallium-68 DOTATATE scan determined significant somatostatin receptor avidity as hypothesized, suggesting that this imaging modality may be used as an option for diagnostic and follow-up purposes in GIST patients. In addition, peptide receptor-mediated radiotherapy (177Lu-PPRT) via SSTR may provide a novel treatment strategy in carefully selected SSTR-avid GIST patients with thyrosine kinase inhibitor (TKI)-resistant tumors such as this case, and this warrants further investigation in novel clinical trial concepts.

## Introduction

This manuscript reports on a patient with a metastatic gastrointestinal stromal tumor (GIST) refractory to standard first-line treatment, who underwent a gallium-68 scan based on pre-clinical data of somatostatin receptor (SSTR) expression in such tumors. The gallium-68 DOTATATE scan determined significant somatostatin receptor avidity as hypothesized, suggesting that this imaging modality may be used as an option for diagnostic and follow-up purposes in GIST patients. This is significant, as it may provide a new target-specific diagnostic option similar to the case of neuroendocrine tumors. In addition, the tumor was noted to be radiosensitive, and peptide receptor-mediated radiotherapy (177Lu-PPRT) via SSTR may provide a novel treatment strategy in carefully selected SSTR-avid GIST patients with thyrosine kinase inhibitor (TKI)-resistant tumors such as this case, which undoubtedly warrants further investigation. Hereby, we report on this interesting patient's case.

## Case presentation

The patient is a 35-year-old male who was in his usual state of health until the summer of  2016, when he started to complain of worsening upper abdominal pain. This led to a visit to the emergency department (ED), and he had a computed tomography (CT) scan of the abdomen and pelvis done, which showed an incidental 3.4 cm rounded hypoattenuation in the right hepatic lobe. A [18F] fluorodeoxyglucose (18F-FDG) positron emission tomography/computed tomography (PET/CT) scan reported a fluorodeoxyglucose (FDG)-avid right retropharyngeal lymph node, hepatic dome lesion, and two osseous lesions in the lumbar vertebra L4 and left proximal sacral ala consistent with hypermetabolic malignancy. Differential considerations included lymphoma, metastatic disease from unknown primary, and less likely primary hepatic malignancy with metastasis.

The patient then underwent a liver biopsy, which reported spindle cell tumor, with equivocal succinate dehydrogenase B (SDHB) expression in the tumor cells; the ERG stain highlighted endothelial cells but was negative for tumor cells, and the mitotic count was 9/50 high power field. The tumor cells were positive for CD117 (KIT) and DOG1, and negative for SOX10, HMB-45, Melan A, desmin, SMA, CD31, ERG, synaptophysin, chromogranin, keratins AE1/AE3, Cam 5.2, arginase, Hep-Par 1, PAX-8, p63, CD138, and CD43. Evaluation of pathology at the National Institutes of Health reported a highly epithelioid neoplasm consistent with succinate dehydrogenase (SDH)-deficient gastrointestinal stromal tumor (GIST). The tumor cells were positive for KIT and SDHA, and negative for SDHB. Next-generation sequencing of the tumor (GenPath, Elmwood Park, NJ) showed no mutations in their panel, including KIT and platelet-derived growth factor A (PDGFRA). We discussed the option of pursuing germline genetic testing for genes associated with SDH-deficient tumors by way of a next-generation sequencing multi-gene panel. After our discussion and given the patient's young age, he elected to pursue Invitae's Hereditary Paraganglioma-Pheochromocytoma Panel (San Francisco, California). The genes included on this panel were: MAX, NF1, RET, SDHA, SDHAF2, SDHB, SDHC, SDHD, TMEM127, and VHL, and the results showed no germline alterations.

We considered the patient to have Stage IV SDH-deficient GIST metastatic to liver and spine, KIT and PDGFRA wild-type, and as a suitable candidate for palliative systemic therapy in order to improve his survival and quality of life. Upper endoscopy was not performed as there was no evidence on imaging of lesions and would not have altered the treatment plan. We discussed that ‘wild-type’ GISTs are usually highly resistant to tyrosine kinase inhibitors (TKIs) but may convey a relatively indolent course [1]. A trial of imatinib was started, with plans of using sunitinib or regorafenib in the event of progression. Restaging the 18F-FDG PET/CT scan 10 weeks later reported interval progression of metastatic disease with both increases in size and standardized uptake value (SUV) in previously seen lesions as well as new osseous metastatic disease. Enhanced magnetic resonance cholangiopancreatography (MRCP) of the abdomen confirmed hepatic and osseous metastases. Osseous metastatic disease is relatively uncommon in GIST; however, it has been reported in several case series [2]. Even though one can argue that the retropharyngeal node and bone metastases could be the primary site of a metachronous paraganglioma, the fast progression of the extensively studied tumor within the liver, along with the development of those lesions, were significantly more consistent with a malignant GIST displaying an aggressive behavior. Regorafenib 160 mg orally on days 1-21 every 28 days and monthly denosumab were started. Guardant360 (Redwood City, California) circulating tumor deoxyribonucleic acid (DNA) assay ‘liquid biopsy’ reported no detectable alterations.

The patient also received palliative fractionated radiation to liver and head and neck (right retropharyngeal mass) from late 2016 through early 2017, concomitant with regorafenib use. A follow-up 18F-FDG PET/CT scan in March 2017 showed an interval decrease in the FDG uptake in the right parapharyngeal mass and right hepatic lobe metastatic lesion, suggestive of partial treatment response status post interval radiation therapy. However, there was a moderate interval increase in the FDG uptake in the multiple osseous metastatic lesions. No new metastatic lesions were noted. After a multidisciplinary meeting and discussion, and given stable disease per response evaluation criteria in solid tumors (RECIST) criteria, the patient continued taking regorafenib accordingly. He was planned for future enrollment in an immune checkpoint inhibitor-based clinical trial [3].

**Figure 1:**
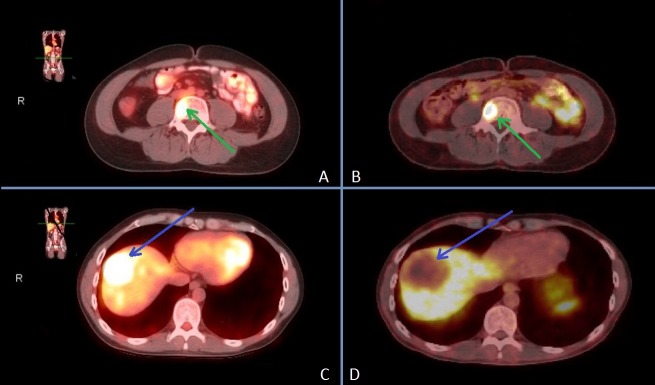
18F-FDG PET/CT and gallium-68 DOTATATE scans showing matched target lesions in liver and bone metastases. It is important to note that the lack of DOTATATE avidity in a circumscribed region of the liver is due to SBRT radiation-induced tumor necrosis A) 18F-FDG PET/CT vertebral body lesion (green arrow) B) 68Ga-DOTATOC SSTR PET/CT vertebral body lesion (green arrow) C) 18F-FDG PET/CT liver lesion prior to SBRT treatment (blue arrow) D) 68Ga-DOTATOC SSTR PET/CT liver lesion after SBRT treatment (blue arrow) 18F-FDG PET/CT: [18F] fluorodeoxyglucose  positron emission tomography/computed tomography SBRT: Stereotactic body radiation therapy SSTR: somatostatin receptor

Given some pre-clinical data regarding the expression of somatostatin receptors (SSTR) in GISTs as a potential target for a therapeutic strategy [4-5], the patient underwent a gallium-68 DOTATATE scan, which reported significant somatostatin receptor avidity in existing lesions as hypothesized (Figure 1 ), suggesting that this imaging modality may be used as an option for diagnostic and follow-up purposes in GIST patients, similar to the indication of  gallium-68 DOTATATE in patients with somatostatin receptor positive neuroendocrine tumors (NETs) [6].

 

Notably, the 18F-FDG PET/CT scan showed a completely matched finding with glucose hypermetabolism of the liver and bone metastases as noted in the gallium-68 DOTATATE scan (Figure 1).

## Discussion

Neuroendocrine tumors' (NETs) overexpression of SSTRs has enabled personalized ‘theranostic’ advances using radiolabeled somatostatin analogues; SSTRs targeting with 177Lu-DOTATATE peptide receptor-mediated radiotherapy (177Lu-PPRT) has demonstrated efficacy and short-term safety, and could be considered for advanced NET patients with disease that express SSRTs and is otherwise refractory to medical therapy [7]. Gallium-68 DOTATATE PET/CT offers utility for diagnosis, prognostication, patient selection, assessment of therapeutic response, and long-term follow-up after peptide receptor radionuclide therapy (PRRT) [8].

Gastrointestinal stromal tumors have been reported to express somatostatin receptors and bind radiolabeled somatostatin analogs, with some authors considering the possibility of using such receptors as a new prognostic biomarker and potential therapeutic strategy [4-5]; however, this is the first report describing the utility of gallium-68 DOTATATE for their detection and to suggest PRRT as a potential treatment option for GIST patients. It is unclear if this biomarker feature is specific to SDH-deficient GIST, or if the presence of somatostatin receptors is a common finding in all types of GIST, which seems very plausible. Lastly, the inclusion of radiotherapy in the management of GIST is considered mostly for palliation, and there is emerging recent data suggestive that the concomitant use of TKIs may improve its efficacy, such as in the present case [9-10]; this was further demonstrated by the significant response to stereotactic body radiation therapy (SBRT) in the liver and the retropharyngeal mass sites reported by 18F-FDG PET/CT.

## Conclusions

Peptide receptor-mediated diagnostics (gallium-68 DOTATATE) and radiotherapy (177Lu-PPRT) via SSTR targeting may provide a novel treatment strategy in carefully selected SSTR-avid GIST patients with TKI-resistant tumors such as this case. In the era of precision medicine and biomarkers, future theranostic studies may consider the inclusion of PRRT with or without TKI in appropriately selected patients; this warrants further investigation in prospective trials.
